# Abnormal Resting-State Functional Connectivity of the Anterior Cingulate Cortex in Unilateral Chronic Tinnitus Patients

**DOI:** 10.3389/fnins.2018.00009

**Published:** 2018-01-23

**Authors:** Yu-Chen Chen, Shenghua Liu, Han Lv, Fan Bo, Yuan Feng, Huiyou Chen, Jin-Jing Xu, Xindao Yin, Shukui Wang, Jian-Ping Gu

**Affiliations:** ^1^Department of Radiology, Nanjing First Hospital, Nanjing Medical University, Nanjing, China; ^2^Department of Radiology, Beijing Friendship Hospital, Capital Medical University, Beijing, China; ^3^Department of Otolaryngology, Nanjing First Hospital, Nanjing Medical University, Nanjing, China; ^4^Department of Clinical Laboratory, Nanjing First Hospital, Nanjing Medical University, Nanjing, China; ^5^Department of Vascular and Interventional Radiology, Nanjing First Hospital, Nanjing Medical University, Nanjing, China

**Keywords:** tinnitus, rostral ACC, dorsal ACC, functional connectivity, resting-state fMRI

## Abstract

**Purpose:** The anterior cingulate cortex (ACC) has been suggested to be involved in chronic subjective tinnitus. Tinnitus may arise from aberrant functional coupling between the ACC and cerebral cortex. To explore this hypothesis, we used resting-state functional magnetic resonance imaging (fMRI) to illuminate the functional connectivity (FC) network of the ACC subregions in chronic tinnitus patients.

**Methods:** Resting-state fMRI scans were obtained from 31 chronic right-sided tinnitus patients and 40 healthy controls (age, sex, and education well-matched) in this study. Rostral ACC and dorsal ACC were selected as seed regions to investigate the intrinsic FC with the whole brain. The resulting FC patterns were correlated with clinical tinnitus characteristics including the tinnitus duration and tinnitus distress.

**Results:** Compared with healthy controls, chronic tinnitus patients showed disrupted FC patterns of ACC within several brain networks, including the auditory cortex, prefrontal cortex, visual cortex, and default mode network (DMN). The Tinnitus Handicap Questionnaires (THQ) scores showed positive correlations with increased FC between the rostral ACC and left precuneus (*r* = 0.507, *p* = 0.008) as well as the dorsal ACC and right inferior parietal lobe (*r* = 0.447, *p* = 0.022).

**Conclusions:** Chronic tinnitus patients have abnormal FC networks originating from ACC to other selected brain regions that are associated with specific tinnitus characteristics. Resting-state ACC-cortical FC disturbances may play an important role in neuropathological features underlying chronic tinnitus.

## Introduction

Tinnitus is an auditory phantom perception like ringing, roaring, or buzzing in ears in the absence of any objective external sounds (Jastreboff, 1990; Lockwood et al., 2002; Wegger et al., 2017). It is reported that there are about a prevalence of 10–15% who experienced tinnitus in adults in United States (Henry et al., 2005; Hall et al., 2011; Langguth et al., 2013). Depression, anxiety, irritability, and sleep disturbances, which often significantly impair the quality of daily life can be observed in chronic tinnitus patients (Reynolds et al., 2004; Langguth et al., 2013; Zeman et al., 2014). Previous studies have suggested that the central nervous system (CNS) may play a major role in the pathophysiology of the tinnitus (Lockwood et al., 2002; Eggermont, 2005; Bartels et al., 2007; Chen et al., 2015a). On the basis of electrophysiological and neuroimaging studies, it has been proposed that tinnitus may be generated from the aberrant neuronal activity in the CNS by a variety of mechanisms such as dysfunctional noise canceling, up-regulation of spontaneous firing rates, increased neural synchrony, increase central noise, tonotopic map reorganization, and aberrant neural connectivity to structures within and/or outside the auditory pathway (Lockwood et al., 1998; Noreña and Eggermont, 2003; Kaltenbach et al., 2005; Noreña and Farley, 2013; Zeng, 2013). However, the exact neuropathological mechanism underlying tinnitus has not yet been fully elucidated.

Among the neural hypothesis, dysfunction in the noise canceling system has been suggested as a potential mechanism for chronic tinnitus (Rauschecker et al., 2010; De Ridder et al., 2012; Song et al., 2015a; Leaver et al., 2016a). Moreover, the anterior cingulate cortex (ACC) or subcallosal ACC or ventromedial prefrontal cortex (vmPFC) have been regarded as core regions involved in the noise canceling system that relates to interoceptive-autonomic processing (Boly et al., 2007; Seeley et al., 2007). The ACC is composed of a number of subregions with different functional significance, within which the rostral and dorsal components are most relevant in tinnitus pathophysiology (Vanneste et al., 2010; Kreuzer et al., 2015; Song et al., 2015a,b; De Ridder et al., 2016). The rostral ACC (rACC) has been shown to be involved in emotion experience and processing while the dorsal ACC (dACC) plays a major role in executive functions by influencing multiple cognitive processes (Bush et al., 2000). Using electroencephalography (EEG) technique, Song et al. suggested the role of the rACC as the core of the descending noise cancellation system, and that dysfunction of the rACC may be relevant with tinnitus perception (Song et al., 2015b). Moreover, Vanneste et al. observed that tinnitus distress is associated with increased alpha and beta activity in the dACC which might be involved in persisting attention to the tinnitus (Vanneste et al., 2010). In addition, Kreuzer et al. used the repetitive transcranial magnetic stimulation (rTMS) with double cone coil to target the ACC for the treatment of patients suffering from chronic tinnitus but failed to show better outcome compared to an actively rTMS treated control group (Kreuzer et al., 2015). In the study of Golm et al. (2013), tinnitus patients and healthy controls underwent functional magnetic resonance imaging (fMRI) while sentences with neutral, negative or tinnitus-related content were presented. They observed that tinnitus patients showed stronger activations to tinnitus-related sentences (e.g., “I will never get rid of the noise”) in comparison to neutral sentences than healthy controls in specific limbic regions such as the ACC, which seem to be involved in the emotional processing of tinnitus-related cognitions (Vanneste et al., 2010). Nonetheless, the role of ACC and the diverse function of ACC rostral and dorsal subregions in tinnitus are still far from clear.

Resting-state fMRI of spontaneous blood oxygenation level-dependent (BOLD) responses has proved to be a useful noninvasive technique to assess the underlying pathogenesis of tinnitus-induced neural dysfunction (Husain and Schmidt, 2014; Chen et al., 2017b). Maudoux et al. detected increased resting-state functional connectivity (FC) between auditory cortices and ACC in tinnitus patients with hearing loss compared to age and sex-matched healthy controls by using connectivity graph analysis (Maudoux et al., 2012b). After featuring a control group that was well-matched for age, sex, and hearing loss, our prior studies found aberrant effective connectivity between the amygdala and ACC in chronic tinnitus patients and decreased amygdala FC with the ACC in depressive tinnitus patients (Chen et al., 2017a,c). However, it remains unclear whether there exist alterations of FC between the ACC and other brain regions in chronic tinnitus patients.

Thus, the ACC seems to be an appropriate location for investigating the FC alterations in patients with chronic tinnitus. In this study, we used a seed-based approach to investigate the intrinsic FC originating from rACC and dACC regions with the whole brain between chronic tinnitus patients and healthy controls. The hypothesis is that (1) resting-state FC characteristics of rACC and dACC regions in tinnitus patients would be significantly different from healthy controls; (2) there exist a clinical relevance between the altered FC and specific tinnitus characteristics (e.g., the tinnitus duration or tinnitus distress).

## Materials and methods

### Subjects

**Figure 1:**
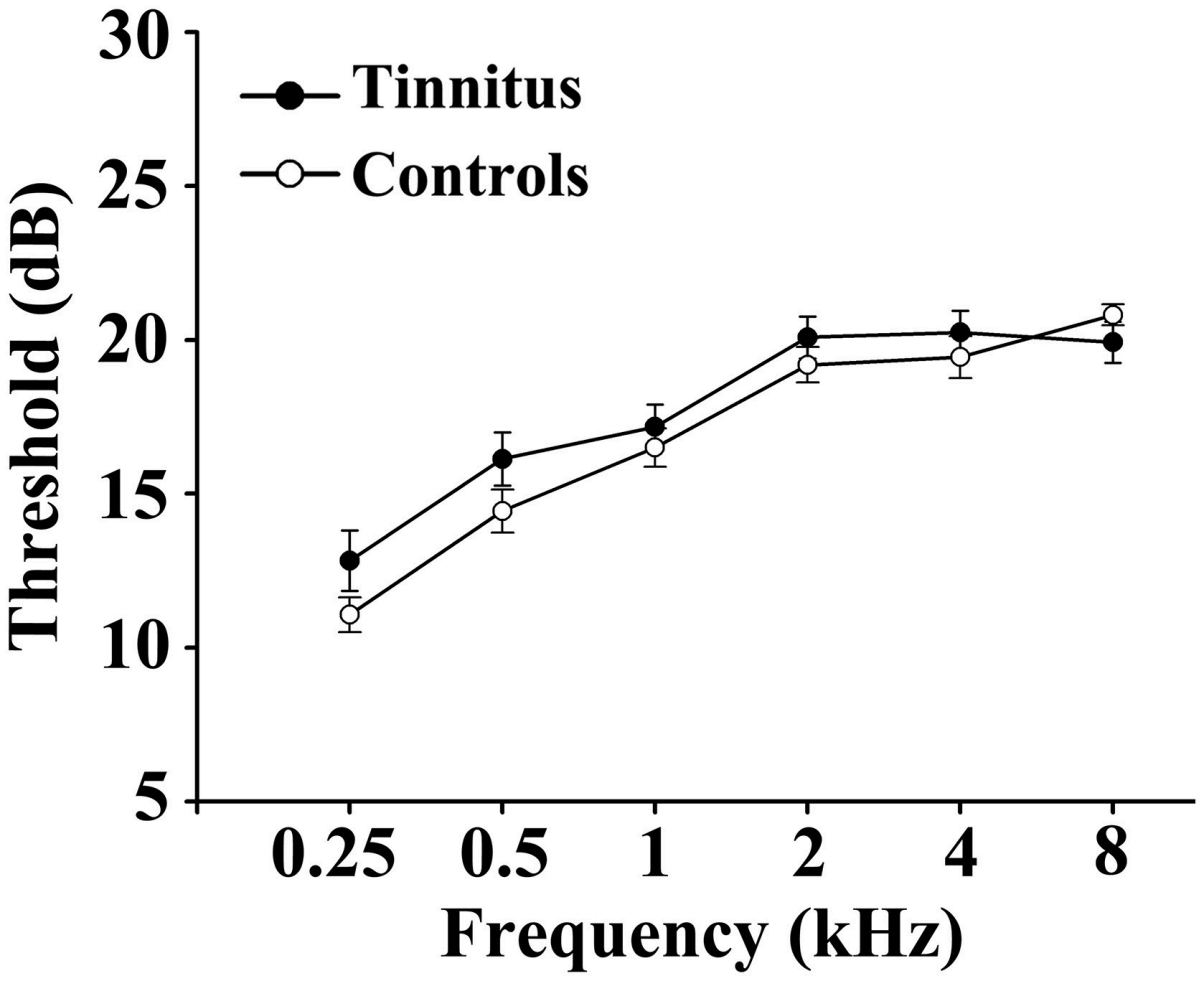
No significant differences in auditory thresholds between tinnitus and control groups. Data are presented as mean ± SEM.

This study included 31 unilateral (all left-sided) tinnitus patients and 40 healthy subjects (all right handed, with at least 8 years of education). The tinnitus subjects were outpatients at the clinic of the Department of Otolaryngology at Nanjing First Hospital. The healthy controls were recruited through community health screening or newspaper advertisement. The patients were group-matched in terms of age, sex, and education. The severity of tinnitus and related distress were assessed by the Iowa version of the Tinnitus Handicap Questionnaires (THQ) (Kuk et al., 1990). The hearing threshold was determined by puretone audiometry (PTA) examination. All the participants had no hearing loss in any of six measured audiometric frequencies ranging from 250 Hz to 8 kHz (hearing thresholds <25 dB). There were no significant differences in auditory thresholds between tinnitus and control groups (Figure 1 and Table 1). No included participants had accompanied symptoms such as depression and anxiety according to the Self-Rating Depression Scale (SDS) and Self-Rating Anxiety Scale (SAS) (overall scores <50, respectively) (Zung, 1971, 1986). According to previous study (Khalfa et al., 2002), we used the Hyperacusis Questionnaire to exclude the participants with hyperacusis in the current study. Partiocipants were excluded if they suffer from Meniere's diseases, pulsatile tinnitus, or hyperacusis, or if they had a past history of severe alcoholism, smoking, head injury, stroke, Alzheimer's disease, Parkinson's disease, epilepsy, major depression, or other neurological or psychiatric illness, major medical illness (e.g., cancer, anemia, and thyroid dysfunction), MRI contraindications, and severe visual loss. Table 1 summarizes the characteristics of the chronic tinnitus patients and healthy subjects. All the participants provided written informed consent before their participation in the study protocol, which was approved by The Research Ethics Committee of the Nanjing Medical University (Reference No. 2016067).

**Table 1:** Characteristics of tinnitus patients and healthy controls.

	**Tinnitus patients (*n* = 31)**	**Healthy controls (*n* = 40)**	***p*-values**
Age (years)	51.4 ± 13.3	48.2 ± 14.2	0.336
Male/Female	14/17	19/21	0.845
Education levels (years)	12.1 ± 2.9	12.2 ± 3.3	0.918
Tinnitus duration (months)	40.6 ± 35.5	–	–
THQ score	51.7 ± 15.8	–	–
SDS score	41.6 ± 4.9	39.9 ± 4.6	0.130
SAS score	39.2 ± 6.7	36.9 ± 5.5	0.114
Gray matter	581.3 ± 25.5	576.5 ± 22.1	0.403
White matter	534.8 ± 24.0	527.6 ± 25.3	0.226
Brain parenchyma	1116.1 ± 31.9	1104.1 ± 35.8	0.147
Hearing thresholds (left)	15.6 ± 3.6	16.8 ± 2.5	0.126
Hearing thresholds (right)	16.9 ± 3.4	17.0 ± 2.2	0.780
Hearing thresholds (average)	16.3 ± 3.0	16.9 ± 1.5	0.267

*Data are represented as Mean ± SD. THQ, Tinnitus Handicap Questionnaire; SDS, Self-Rating Depression Scale; SAS, Self-Rating Anxiety Scale*.

### MRI scanning

All subjects were scanned using a 3.0 T MRI scanner (Ingenia, Philips Medical Systems, Netherlands) with a standard head coil. Head motion and scanner noise were reduced using foam padding and earplugs. The earplugs (Hearos Ultimate Softness Series, USA) were used to attenuate scanner noise by approximately 32 dB. No subjects found the scanner noise uncomfortable during the scanning. The subjects were instructed to lie quietly with their eyes closed but not to fall asleep, and avoid thinking of anything particular during the scanning. Structural images were acquired with a three-dimensional turbo fast echo (3D-TFE) T1WI sequence with high resolution as follows: TR/TE = 8.1/3.7 ms; slices = 170; thickness = 1 mm; gap = 0 mm; FA = 8°; acquisition matrix = 256 × 256; FOV = 256 × 256 mm. The structural sequence took 5 min and 29 s. Functional images were obtained axially using a gradient echo-planar imaging sequence as follows: repetition time (TR) = 2,000 ms; echo time (TE) = 30 ms; slices = 36; thickness = 4 mm; gap = 0 mm; field of view (FOV) = 240 × 240 mm; acquisition matrix = 64 × 64; and flip angle (FA) = 90°. The fMRI sequence took 8 min and 8 s.

### Data preprocessing

Data analyses were preprocessed using Data Processing Assistant for Resting-State fMRI programs (Chao-Gan and Yu-Feng, 2010), which is based on Statistical Parametric Mapping (SPM8, http://www.fil.ion.ucl.ac.uk/spm) and resting-state fMRI data analysis toolkit (REST, http://www.restfmri.net). The first 10 volumes were discarded and the remaining 230 consecutive volumes were used for data analysis. Slice-timing and realignment for head motion correction were performed. Any subjects with a head motion >2.0 mm translation or a 2.0° rotation in any direction were excluded. According to the criteria, no participants were excluded from the analysis due to head motion in this study. After that, spatial normalization to the Montreal Neurological Institute template (resampling voxel size = 3 × 3 × 3 mm^3^), smoothing with an isotropic Gaussian kernel [full width at half maximum (FWHM) = 6 mm], detrending and filtering (0.01–0.08 Hz) were performed in order.

### Structural data analysis

Voxel-based morphometry (VBM) approach was performed to compute the gray matter (GM) volume and white matter (WM) volume of each subject based on the VBM8 toolbox (http://dbm.neuro.uni-jena.de/vbm). Briefly, cerebral tissues were segmented into GM, WM, and cerebrospinal fluid and were then normalized to the MNI space using a unified segmentation algorithm (Ashburner and Friston, 2005). T1 images were normalized to the MNI template using affine linear registration followed by Gaussian smoothing (FWHM = 6 mm). GM and WM volumes were calculated by estimating these segments. Brain parenchyma volume was calculated as the sum of GM and WM volumes.

### Functional data analysis

The regions of interest (ROIs) were generated from Brodmann template by selecting the rACC and dACC as seed regions using the WFU_PickAtlas software (http://www.ansir.wfubmc.edu). The mean time series of each ROI was acquired for reference time course. Pearson's correlation coefficients were then computed between the mean signal change of each ROI and the time series of each voxel. Finally, the correlation coefficients were converted into *z*-values using Fisher z-transform to improve the normality (Lowe et al., 1998). Six parameters of head motion and average time courses of global, WM, and CSF signals were removed by linear regression analysis.

For within-group analysis, the individual *z*-values were entered into the SPM8 software for a random effect one-sample *t*-test to determine the brain regions showing significant connectivity to each ROI at a threshold of *p* < 0.01 with multiple comparisons correction using the false discovery rate (FDR) criterion. Two-sample *t*-tests were performed to identify FC differences in each ROI between tinnitus patients and controls within a default GM mask. Age, sex, education, GM volume, and average hearing thresholds were included as nuisance covariates. The significance of group differences was set at a threshold of *p* < 0.01 [cluster-level family-wise error (FWE) correction].

### Statistical analysis

Differences in demographic data between tinnitus patients and healthy controls were analyzed using between-group *t*-test for means and χ^2^-test for proportions (*p* < 0.05 was considered to be significant). To investigate the relationship between fMRI data and clinical characteristic of tinnitus patients, regions showing significant differences between groups were extracted. Then the mean *z*-values of aberrant FC region mask were calculated within every subject. Pearson correlation analysis between the mean *z*-values and each clinical characteristic were performed using SPSS 19.0 (version 19.0; SPSS, Chicago, IL, USA). *p* < 0.05 was considered statistically significant. Partial correlations were calculated after correction for age, sex, education, GM volume, and average hearing thresholds. Bonferroni correction was used for multiple comparisons in the correlation analyses.

## Results

### Structural analysis

Comparisons of the whole brain volumes (GM volume, WM volume, and brain parenchyma volume) between the unilateral tinnitus patients and healthy controls were presented in Table 1. No significant differences in GM and WM volumes were found between tinnitus patients and the control group (*p* > 0.05).

### Functional analysis

**Figure 2:**
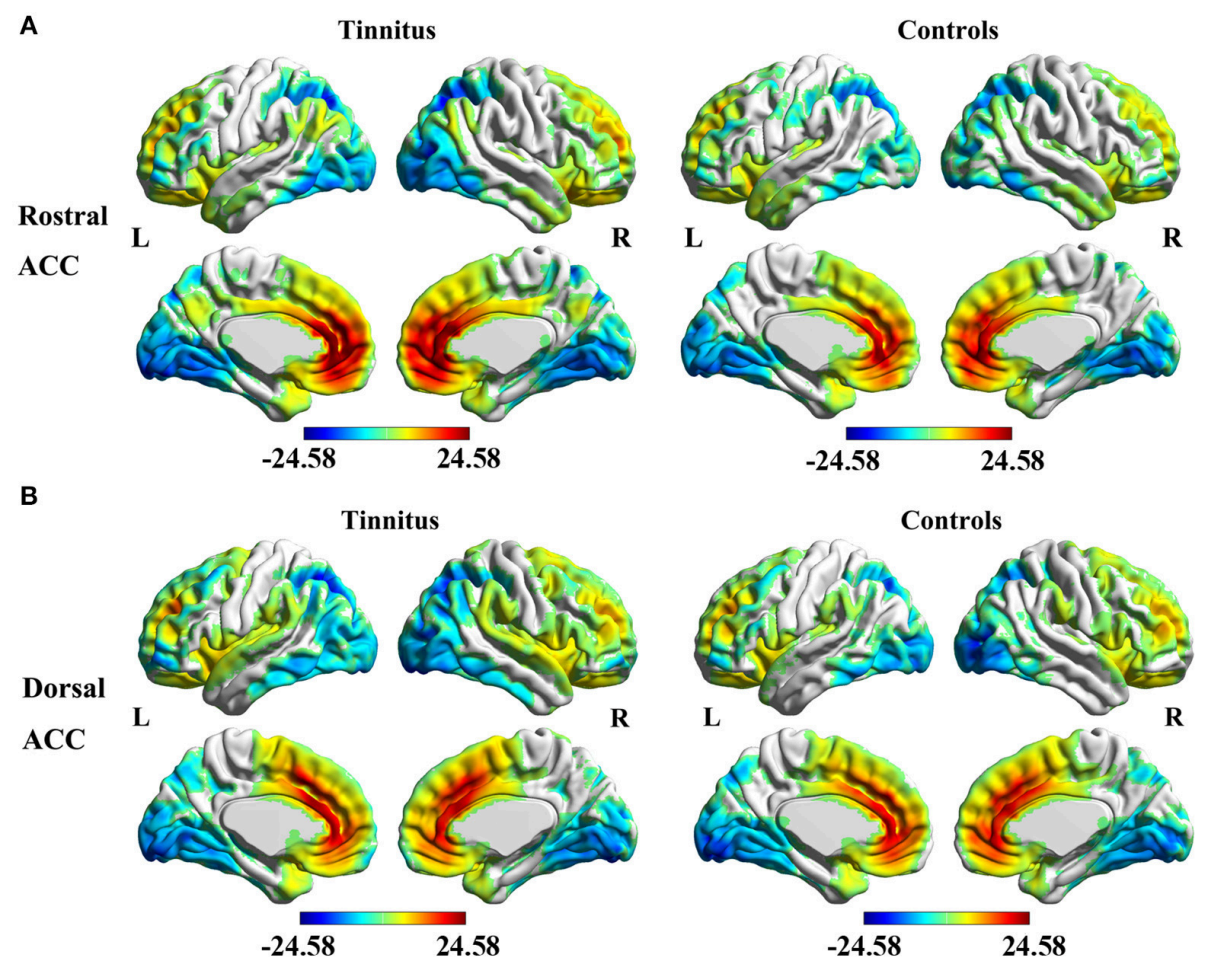
Significant FC of the rostral ACC **(A)** and the dorsal ACC **(B)** in whole brain using one-sample *t*-test in both unilateral tinnitus patients and healthy controls. Significant thresholds were corrected using cluster-level family-wise error (FWE) criterion and set at *p* < 0.01.

The analyses of one-sample *t*-test revealed the rACC (Figure 2A) and dACC (Figure 2B) FC maps in both tinnitus patients and healthy controls. The rACC and dACC mainly exhibited positive FC with the medial prefrontal gyrus, superior temporal gyrus (STG), parietal, and cingulate cortex. In contrast, the inferior temporal gyrus and occipital cortex showed negative FC with the rACC and dACC.

**Figure 3:**
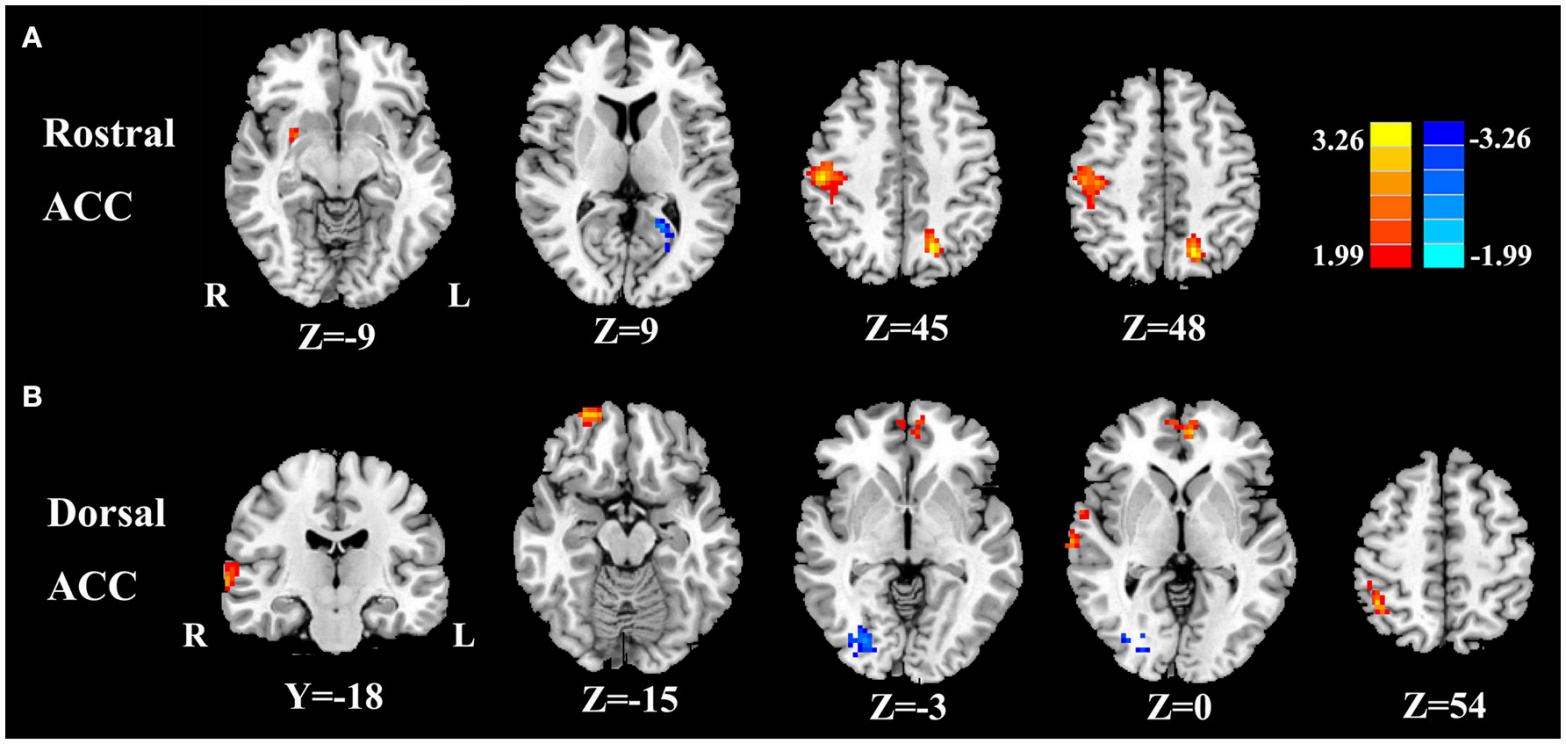
Aberrant FC of the rostral ACC **(A)** and the dorsal ACC **(B)** in unilateral tinnitus patients compared with healthy controls. The threshold was set at a *p* < 0.01 (FWE corrected). Note that the left side corresponds to the right hemisphere.

Compared with healthy controls, unilateral tinnitus patients showed significantly increased FC between the rACC and left precuneus, right postcentral gyrus, and right putamen as well as decreased FC with the left calcarine cortex (Figure 3A and Table 2, *p* < 0.01, FWE corrected). Furthermore, unilateral tinnitus patients relative to controls also demonstrated significantly increased FC between the dACC and right STG, right inferior parietal lobule (IPL), right orbitofrontal cortex (OFC), and right medial prefrontal gyrus as well as reduced FC with the fusiform gyrus (Figure 3B and Table 2, *p* < 0.01, FWE corrected).

**Table 2:** Abnormal functional connectivity of rostral and dorsal ACC in unilateral tinnitus patients compared to healthy controls.

**Brain region**	**BA**	**MNI Coordinates x, y, z (mm)**	**T score**	**Voxels**
**ROSTRAL ACC**				
L precuneus	7	−18, −66, 48	3.4455	85
R postcentral gyrus	2	51, −21, 45	3.4431	164
R putamen	–	27, 3, −9	2.5116	45
L calcarine cortex	19	−21, −54, 9	−3.1271	40
**DORSAL ACC**				
R superior temporal gyrus	21	69, −18, 0	3.7365	85
R inferior parietal lobule	40	42, −51, 54	3.0380	79
R orbitofrontal cortex	11	21, −60, −15	3.1685	44
R medial prefrontal gyrus	10	−6, 51, 0	3.1812	50
R fusiform gyrus	18	27, −81, −3	−3.7313	56

*The threshold was set at a p < 0.01 (FWE corrected). BA, Brodmann's area; MNI, Montreal Neurological Institute; L, left; R, right*.

### Correlation analysis results

**Figure 4:**
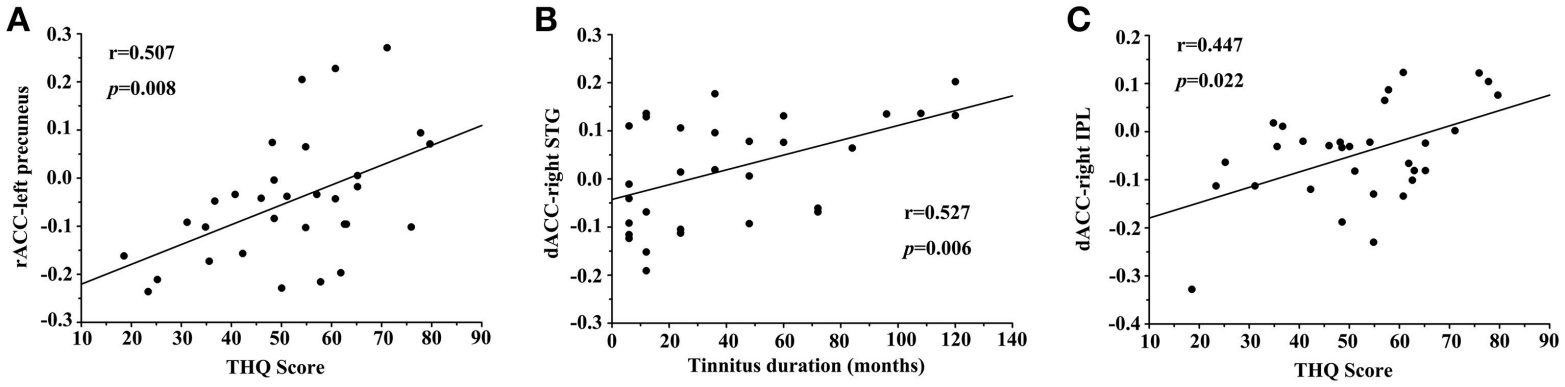
Significant correlations between the abnormal FC and tinnitus characteristics. **(A)** Correlation between the increased FC of the rACC-left precuneus and the THQ score; **(B)** Correlation between the increased FC of the dACC-right STG and the tinnitus duration; **(C)** Correlation between the increased FC of the dACC-right IPL and the THQ score.

In unilateral tinnitus patients, the increased FC between the rACC and left precuneus was positively correlated with the THQ score (*r* = 0.507, *p* = 0.008) (Figure 4A). Furthermore, the increased FC between the dACC and right STG was positively correlated with the tinnitus duration (*r* = 0.527, *p* = 0.006) (Figure 4B). In addition, the increased FC between the dACC and right IPL was positively correlated with the THQ score (*r* = 0.447, *p* = 0.022) (Figure 4C). These correlations had been corrected for age, sex, education, GM volume, and average hearing thresholds. Other measures of increased or decreased FC were independent of tinnitus duration or THQ score. None of the disrupted FC was correlated with SAS or SDS score. Nevertheless, no significant correlations persisted after Bonferroni correction, probably partly due to the relatively strict calculation.

## Discussion

This is the first study exploiting the aberrant FC of the rACC and dACC regions to provide novel insight into the underlying neural mechanisms of unilateral chronic tinnitus. We found that chronic tinnitus changed the FC pattern within several ACC-cortical networks, including the auditory cortex, prefrontal cortex, visual cortex, and default mode network (DMN). Moreover, disrupted FC originating from the ACC subregions in tinnitus patients was positively correlated with the specific tinnitus characteristics like tinnitus duration and tinnitus distress.

Structural alterations could conceivably contribute to these functional abnormalities. However, the current study did not detect any brain structural differences between our normal hearing tinnitus patients and matched healthy controls, which was consistent with our previous studies (Chen et al., 2014, 2015b,d, 2017c). Although decreased or increased GM changes in several brain regions of tinnitus patients have been reported from previous research, the changes in GM volume seen in these tinnitus patients were typically correlated with hearing loss particularly when testing was extended out beyond 8 kHz (Leaver et al., 2012; Seydell-Greenwald et al., 2012; Boyen et al., 2013, 2014). Moreover, the heterogeneity of the tinnitus population and the MR analytical method may contribute to the differences. Nonetheless, our results may suggest that abnormal FC can exist prior to major structural alterations in tinnitus patients with normal hearing.

In our study, the right STG showed increased FC to the dACC subregion, which plays a crucial role in a form of attention regulating emotional and cognitive functions and therefore could be important as an emotional and attentional regulator of tinnitus (Bush et al., 2000). Prior EEG suggests that the dACC might be involved in persistent attention to tinnitus (Vanneste et al., 2010; De Ridder et al., 2011; Song et al., 2013, 2015a,b). However, Song et al. reported a negative correlation of the ACC activity and its connectivity to the auditory cortex with the tinnitus awareness percentage in tinnitus patients (Song et al., 2015a), which was inconsistent with our increased FC results. The discrepancy between studies might be due to the different neuroimaging methods used to investigate tinnitus (EEG vs. fMRI) and the heterogeneity of tinnitus patients (bilateral tinnitus vs. unilateral tinnitus) (Vanneste et al., 2011). Furthermore, the positive correlation of FC between the right STG and the dACC with the tinnitus duration has been demonstrated, suggesting that the interaction between ACC and auditory cortex may be involved in the neuropathological changes in chronic tinnitus. An alternative hypothesis is that the tinnitus percept is derived from disordered activity among several auditory and non-auditory regions (Horwitz and Braun, 2004; Husain et al., 2006). A gating model is presented by Rauschecker et al. in which tinnitus is a result of a failure to prevent the noise signals to reach the auditory center (Rauschecker et al., 2010). And Leaver et al. found that the aberrant brain network may be the precondition of the auditory-sensory experience of tinnitus (Leaver et al., 2016b). Therefore, the abnormal increase of FC between the auditory cortex and the ACC may be constituent parts of the pathological conditions of tinnitus for creating or failing to filter the unpleasant sound in the auditory pathway.

The prefrontal cortices that are associated with executive functions, including the medial prefrontal cortex, showed increased FC to the dACC in our current study. The vital role for the prefrontal cortex in subserving tinnitus mechanism has been postulated and previous fMRI studies have suggested the aberrant coupling between the prefrontal cortex and other regions for tinnitus (Lanting et al., 2009; Rauschecker et al., 2010; Leaver et al., 2011; Seydell-Greenwald et al., 2012; Chen et al., 2014, 2015d, 2017b,c). Rauschecker et al. developed a model to demonstrate structural and functional differences in vmPFC between tinnitus patients and controls. Importantly, the observed neuronal response in vmPFC was correlated with the subjective loudness, indicating that prefrontal cortex may contribute to certain perceptual features of tinnitus (Rauschecker et al., 2010). Moreover, resting-state fMRI studies have also suggested the abnormal FC from the OFC in chronic tinnitus (Maudoux et al., 2012b; Chen et al., 2015c; Zhang et al., 2015). The OFC is regarded as part of the reward system, which might integrate the aversive information of the perceived tinnitus (Rolls, 2004; Kringelbach, 2005). The heightened FC between the ACC and the OFC might be interpreted as a dysfunctional inhibitory response directing attention away from phantom sound perception. Our finding of positive functional coupling between the ACC and the OFC may also imply the possible role of the OFC in tinnitus.

Furthermore, our tinnitus patients showed increased FC between the rACC and the precuneus as well as the dACC and the IPL, which exhibited positive correlations with the THQ scores. Precuneus is one of the key brain areas which are associated with tinnitus distress (Husain and Schmidt, 2014; Husain, 2016; Pattyn et al., 2016). The precuneus and IPL belong to the DMN, which is most active at rest and shows reduced activity when a subject enters a task-based state involving attention or goal-directed behavior (Raichle et al., 2001; Mantini et al., 2007). Prior resting-state fMRI studies have also found aberrant FC networks within the DMN in tinnitus patients compared with healthy controls (Burton et al., 2012; Schmidt et al., 2013, 2017; Chen et al., 2014, 2015b, 2017c; Lanting et al., 2016). However, the source of disrupted neuronal activity within specific DMN regions resulting from tinnitus still remains unknown. Our results suggest that increased FC patterns of precuneus and IPL might be responsible for disrupting the DMN in tinnitus patients.

Our current study observed increased connectivity between the postcentral gyrus and the ACC in phantom tinnitus perceptions. Possible neural correlates of somatosensory modulation of tinnitus were assessed (Murray et al., 2004), which was in line with prior fMRI studies showing aberrant neural activity in somatosensory networks in tinnitus (Smits et al., 2007; Maudoux et al., 2012a; Chen et al., 2017c). Moreover, increased FC of the subcortical structure such as the putamen, has also been suggested to be involved in tinnitus perception through fMRI studies (Hinkley et al., 2015; Rauschecker et al., 2015; Leaver et al., 2016b). Interestingly, the occipital cortex that is associated with the visual recognition including the calcarine cortex and fusiform gyrus showed reduced FC with the ACC in this study. Consistently, fMRI studies also revealed decreased neural activity and connectivity in visual cortex in chronic tinnitus (Burton et al., 2012; Chen et al., 2014, 2015c). One possibility is due to compensatory mechanisms in visual regions associated with hearing a phantom sound. It has been suggested that sensory deprivation in the auditory modality could affect the function of the visual modality (Bavelier et al., 2006; Dieterich et al., 2007). Moreover, altered attention (shifted toward the auditory modality) could be another explanation but the attention was not controlled or assessed in the current study.

Surprisingly, almost all the abnormal FC patterns especially for the dACC were lateralized to the right hemisphere region in right-sided tinnitus patients. Asymmetry for the tinnitus patients has been reported both structurally and functionally (Smits et al., 2007; Landgrebe et al., 2009; Chen et al., 2014; Geven et al., 2014; Lanting et al., 2014; Lv et al., 2017). Prior PET studies have revealed higher resting-state metabolic activity in right associative auditory brain areas (Geven et al., 2014). Moreover, Chen et al. observed that all the increased spontaneous neural activities were localized in the right hemisphere region in chronic tinnitus (Chen et al., 2014). Nonetheless, several brain regions were detected to be lateralized to the left hemisphere region in our right-sided tinnitus patients, probably indicating that tinnitus not only involves aberrant neural activity but also interaction with other regions of whole brain. Other studies also demonstrated the left-lateralization effect due to tinnitus (Arnold et al., 1996; Langguth et al., 2006; Schecklmann et al., 2013). Therefore, future studies are required to determine if the observed right-lateralization effect is related specifically to tinnitus or some other factors.

Several constraints must be acknowledged in the current study. First, a larger sample size will enhance the reliability of this study. More extensive and longitudinal investigations are required to detect the relationships between aberrant FC patterns of ACC and tinnitus characteristics in further study. Second, subtypes of different etiology, age, severity, and other factors that could produce a deviation of subjective chronic tinnitus need to be divided to reduce the inconsistency of these subgroups. Third, we have to admit that no significant results persisted after the use of a liberal primary threshold (*p* < 0.01) and no Bonferonni correction, probably partly due to the relatively strict calculation. A more stringent threshold (e.g., *p* < 0.001) and Bonferonni correction will be considered in further study. Nonetheless, our research is still meaningful to provide some enlightenments for future study in this field. Moreover, we only selected the rACC and dACC as seed regions to investigate the intrinsic FC patterns with the whole brain of tinnitus. The current seed-based approach could be extended to other non-auditory brain areas involved in tinnitus, such as posterior cingulate cortex (PCC), precuneus, and insula. Additionally, we cannot completely prevent participants from hearing some scanner noise although this study has attempted to minimize the noise with earplugs. The existence of scanner noise may make the internal sound of tinnitus less salient thereby reducing the differences in resting-state networks between tinnitus and control groups. This limitation should be taken into account when interpreting the resting-state fMRI data in auditory related researches. Furthermore, we did not ask the participants to focus on their attention during MR scanning to avoid activating resting-state attention networks of the subjects. We acknowledge that the focused attention or level of distress in subjects will influence the resting-state activity during MR scanning (Damoiseaux et al., 2006; Sonuga-Barke and Castellanos, 2007; Kounios et al., 2008; Logothetis et al., 2009; Husain and Schmidt, 2014). In this way, the differences in attention and distress of the patients might also cause resting-state brain activation and influence our FC measures, not specific to tinnitus itself. Finally, besides the functional disruptions, more researches are required to explore the possibility of structural connectivity in the ACC, which can be measured by the diffusion tensor imaging (DTI).

## Conclusions

In spite of these limitations, our current study identified for the first time abnormal resting-state FC patterns of ACC with several brain structures in unilateral chronic tinnitus patients, including the auditory cortex, prefrontal cortex, visual cortex, and some regions of DMN. In addition, disrupted FC was associated with the perception of tinnitus and tinnitus distress. These findings mainly explicated the possible role of ACC and the potential cross-modal neural interaction in tinnitus patients, which may lead to a better understanding of the pathophysiology underlying chronic tinnitus.

## Author contributions

Y-CC and SL: designed the experiment, collected the data, performed the analysis, and wrote the paper; HL, FB, YF, HC, J-JX, and XY: helped collect the data and perform the analysis; J-PG and SW: contributed to the discussion and manuscript revision.

### Conflict of interest statement

The authors declare that the research was conducted in the absence of any commercial or financial relationships that could be construed as a potential conflict of interest.
